# Asymptotic theory for maximum likelihood estimates in reduced-rank multivariate generalized linear models

**DOI:** 10.1080/02331888.2018.1467420

**Published:** 2018-05-08

**Authors:** E. Bura, S. Duarte, L. Forzani, E. Smucler, M. Sued

**Affiliations:** a Institute of Statistics and Mathematical Methods in Economics, TU Wien, Vienna, Austria; b Department of Statistics, George Washington University, Washington, DC, USA; c Facultad de Ingeniería Química, UNL, Santa Fe, Argentina; d Department of Statistics, University of British Columbia, Vancouver, BC, Canada; e Instituto de Cálculo, UBA, Buenos Aires, Argentina

**Keywords:** M-estimation, exponential family, rank restriction, non-convex, parameter spaces

## Abstract

Reduced-rank regression is a dimensionality reduction method with many applications. The asymptotic theory for reduced rank estimators of parameter matrices in multivariate linear models has been studied extensively. In contrast, few theoretical results are available for reduced-rank multivariate generalized linear models. We develop M-estimation theory for concave criterion functions that are maximized over parameter spaces that are neither convex nor closed. These results are used to derive the consistency and asymptotic distribution of maximum likelihood estimators in reduced-rank multivariate generalized linear models, when the response and predictor vectors have a joint distribution. We illustrate our results in a real data classification problem with binary covariates.

## 1. Introduction

The multivariate multiple linear regression model for a *q*-dimensional response $Y=(Y_{1},\ldots ,Y_{q}{)}^{T}$ and a *p*-dimensional predictor vector $X=(X_{1},\ldots ,X_{p}{)}^{T}$ postulates that Y=BX+ϵ, where *B* is a q×p matrix and $\epsilon =(\epsilon_{1},\ldots ,\epsilon_{q}{)}^{T}$ is the error term, with E(ϵ)=0 and var(ϵ)=Σ. Based on a random sample $\left(X_{1},Y_{1}),\ldots ,(X_{n},Y_{n}\right)$ satisfying the model, ordinary least squares estimates the parameter matrix *B* by minimizing the squared error loss function $\sum_{i=1}^{n}∥Y_{i}-BX_{i}{∥}^{2}$ to obtain

(1) $${\hat{B}}_{\mathrm{ols}}=\left(\sum_{i=1}^{n}Y_{i}X_{i}^{T}\right){\left(\sum_{i=1}^{n}X_{i}X_{i}^{T}\right)}^{-1}.$$

Reduced-rank regression introduces a rank constraint on *B*, so that Equation (1) is minimized subject to the constraint rank(B)≤r, where r<min(p,q). The solution is ${\hat{B}}_{\mathrm{RRR}}^{T}={\hat{B}}_{\mathrm{ols}}^{T}U_{r}U_{r}^{T}$, where $U_{r}$ are the first *r* singular vectors of ${\hat{Y}}^{T}={\hat{B}}_{\mathrm{ols}}X^{T}$ (see, e.g. [1]).

Reduced-rank regression has attracted attention as a regularisation method by introducing a shrinkage penalty on *B*. Moreover, it is used as a dimensionality reduction method as it constructs latent factors in the predictor space that explain the variance of the responses.

Anderson [2] obtained the likelihood-ratio test of the hypothesis that the rank of *B* is a given number and derived the associated asymptotic theory under the assumption of normality of *Y* and non-stochastic *X*. Under the assumption of joint normality of *Y* and *X*, Izenman [3] obtained the asymptotic distribution of the estimated reduced-rank regression coefficient matrix and drew connections between reduced-rank regression, principal component analysis and correlation analysis. Specifically, principal component analysis coincides with reduced-rank regression when *Y* =*X* [4]. Recently, Fan et al. [5] studied the theoretical properties of nuclear norm regularized maximum likelihood type estimates in a class of models that includes reduced-rank regression. They derive statistical rates of convergence in possibly high-dimensional scenarios. However, they do not provide asymptotic results that can be used to construct confidence intervals or hypothesis tests. The monograph by Reinsel and Velu [1] contains a comprehensive survey of the theory and history of reduced rank regression, including in time series, and its many applications.

Despite the application potential of reduced-rank regression, it has received limited attention in generalized linear models. Yee and Hastie [6] were the first to introduce reduced-rank regression to the class of multivariate generalized linear models, which covers a wide range of data types for both the response and predictor vectors, including categorical data. Multivariate or vector generalized linear models (VGLMs) is the topic of Yee's [7] book, which is accompanied by the associated R packages, VGAM and VGAMdata. Yee and Hastie [6] proposed an alternating estimation algorithm, which was shown to result in the maximum likelihood estimate of the parameter matrix in reduced-rank multivariate generalized linear models by Bura et al. [8]. Asymptotic theory for the restricted rank maximum likelihood estimates of the parameter matrix in multivariate GLMs has not been developed yet.

In general, a maximum likelihood estimator is a concave M-estimator in the sense that it maximizes the empirical mean of a concave criterion function. Asymptotic theory for M-estimators defined through a concave function has received much attention. Huber [9], Haberman [10] and Niemiro [11] are among the classical references. More recently, Hjort and Pollard [12] presented a unified framework for the statistical theory of M-estimation for convex criterion functions that are minimized over open convex sets of a Euclidean space. Geyer [13] studied M-estimators restricted to a closed subset of a Euclidean space.

The rank restriction in reduced rank regression imposes constraints that have not been studied before in M-estimation as they result in neither convex nor closed parameter spaces. In this paper we (a) develop M-estimation theory for concave criterion functions, which are maximized over parameters spaces that are neither convex nor closed, and (b) apply the results from (a) to obtain asymptotic theory for reduced rank regression estimators in generalized linear models. Specifically, we derive the asymptotic distribution and properties of maximum likelihood estimators in reduced-rank multivariate generalized linear models where both the response and predictor vectors have a joint distribution. The asymptotic theory we develop covers reduced-rank regression for linear models as a special case. We show the improvement in inference the asymptotic theory offers via analysing the data set Yee and Hastie [6] analysed.

Throughout, for a function $f:R^{q}\to R$, ∇f(x) denotes the row vector $\nabla f(x)=(\partial f(x)/\partial x_{1},\ldots ,\partial f(x)/\partial x_{q})$, $\dot{f}(x)$ stands for the column vector of derivatives, while $\nabla^{2}f(x)$ denotes the symmetric matrix of second-order derivatives. For a vector valued function $f:R^{q_{1}}\to R^{q_{2}}$, ∇f denotes the $q_{2}\times q_{1}$ matrix,

$$\nabla f(x)=\left(\begin{array}{cccc} \frac{\partial f_{1}}{\partial x_{1}} & \frac{\partial f_{1}}{\partial x_{2}} & \cdots & \frac{\partial f_{1}}{\partial x_{q_{1}}}\\ \frac{\partial f_{2}}{\partial x_{1}} & \frac{\partial f_{2}}{\partial x_{2}} & \cdots & \frac{\partial f_{2}}{\partial x_{q_{1}}}\\ \vdots & \vdots & \vdots & \vdots \\ \frac{\partial f_{q_{2}}}{\partial x_{1}} & \frac{\partial f_{q_{2}}}{\partial x_{2}} & \cdots & \frac{\partial f_{q_{2}}}{\partial x_{q_{1}}} \end{array}\right).$$

## 2. M-estimators

Let *Z* be a random vector taking values in a measurable space Z and distributed according to the law *P*. We are interested in estimating a finite dimensional parameter $\xi_{0}=\xi_{0}(P)$ using *n* independent and identically distributed copies $Z_{1},Z_{2},\ldots ,Z_{n}$ of *Z*. In the sequel, we use *Pf* to denote the mean of f(Z); i.e. Pf=E[f(Z)].

Let Ξ be a subset of a Euclidean space and $m_{\Xi }:Z\to R$ be a known function. Assume that the parameter of interest $\xi_{0}$ is the maximizer of the map $\xi \mapsto Pm_{\xi }$ defined on Ξ. One can estimate $\xi_{0}$ by maximizing an empirical version of the optimization criterion. Specifically, given a known function $m_{\xi }:Z\mapsto R$, define

(2) $$M(\xi ):=Pm_{\xi }\;\text{and}\;M_{n}(\xi ):=P_{n}m_{\xi },$$

where, here and throughout, $P_{n}$ denotes the empirical mean operator $P_{n}V=n^{-1}\sum_{1=1}^{n}V_{i}$. Hereafter, $M_{n}(\xi )$ and $P_{n}m_{\xi }$ will be used interchangeably as the criterion function, depending on which of the two is appropriate in a given setting.

Assume that $\xi_{0}$ is the unique maximizer of the deterministic function *M* defined in Equation (2). An *M-estimator* for the criterion function $M_{n}$ over Ξ is defined as

(3) $${\hat{\xi }}_{n}={\hat{\xi }}_{n}(Z_{1},\ldots ,Z_{n})\;\text{maximizing}\;M_{n}(\xi )=\frac{1}{n}\sum_{i=1}^{n}m_{\xi }(Z_{i})\;\text{over }\Xi \;.$$

If the maximum of the criterion function $M_{n}$ over Ξ is not attained but the supremum of $M_{n}$ over Ξ is finite, any value ${\hat{\xi }}_{n}$ that almost maximizes the criterion function, in the sense that it satisfies

(4) $$M_{n}({\hat{\xi }}_{n})\geq \underset{\xi \in \Xi }{\sup}M_{n}(\xi )-A_{n},$$

for $A_{n}$ small, can be used instead.

Definition 2.1 An estimator ${\hat{\xi }}_{n}$ that satisfies Equation (4) with $A_{n}=o_{p}(1)$ is called a weak M-estimator for the criterion function $M_{n}$ over Ξ. When $A_{n}=o_{p}(n^{-1})$, ${\hat{\xi }}_{n}$ is called a strong M-estimator.

Proposition A.1 in the Appendix lists the conditions for the existence, uniqueness and strong consistency of an M-estimator, as defined in Equation (3), when $M_{n}$ is concave and the parameter space is convex. Under regularity conditions, as those stated in Theorem 5.23 in van der Vaart [14], the asymptotic expansion and distribution of a consistent strong M-estimator [see Definition 2.1] for $\xi_{0}$ is given by

(5) $$\sqrt{n}({\hat{\xi }}_{n}-\xi_{0})=\frac{1}{\sqrt{n}}\sum_{i=1}^{n}IF_{\xi_{0}}(Z_{i})+o_{p}(1),$$

where $IF_{\xi_{0}}(Z_{i})=-V_{\xi_{0}}^{-1}{\dot{m}}_{\xi_{0}}(Z_{i})$, and $V_{\xi_{0}}$ is the non-singular symmetric second derivative matrix of M(ξ) at $\xi_{0}$.

### 2.1. Restricted M-estimators

We now consider the optimization of $M_{n}$ over $\Xi^{\mathrm{res}}\subset \Xi$, where $\Xi^{\mathrm{res}}$ is the image of a function that is not necessarily injective. Specifically, we restrict the optimization problem to the set $\Xi^{\mathrm{res}}$ by requiring:

Condition 2.2 There exists an open set $\Theta \subset R^{q}$ and a map g:Θ→Ξ such that $\xi_{0}\in g(\Theta )=\Xi^{\mathrm{res}}$.

Even when an M-estimator for the unrestricted problem as defined in (3) exists, there is no a priori guarantee that the supremum is attained when considering the restricted problem. Nevertheless, Lemma 2.3 establishes the existence of a restricted strong M-estimator, regardless of whether the original M-estimator is a real maximizer, weak or strong. All proofs are provided in the Appendix.

Lemma 2.3 Assume there exists a weak/strong M-estimator ${\hat{\xi }}_{n}$ for the criterion function $M_{n}$ over Ξ. If Condition 2.2 holds, then there exists a strong M-estimator ${\hat{\xi }}_{n}^{\mathrm{res}}$ for the criterion function $M_{n}$ over $\Xi^{\mathrm{res}}$.

Proposition 2.4 next establishes the existence and consistency of a weak restricted M-estimator sequence when $m_{\xi }(z)$ is a concave function in *ξ* under the same assumptions as those of the unrestricted problem, stated in Proposition A.1 in the Appendix.

Proposition 2.4 Assume that Condition 2.2 holds. Then, under the assumptions of Proposition A.1 in the Appendix, there exists a strong M-estimator of the restricted problem over $\Xi^{\mathrm{res}}$. Moreover, any strong M-estimator of the restricted problem converges to $\xi_{0}$ in probability.

We derive next the asymptotic distribution of ${\hat{\xi }}_{n}^{\mathrm{res}}=g({\hat{\theta }}_{n})$, with ${\hat{\theta }}_{n}\in \Theta$. The constrained estimator ${\hat{\xi }}_{n}^{\mathrm{res}}$ is well defined under Lemma 2.3, even when ${\hat{\theta }}_{n}$ is not uniquely determined. If ${\hat{\theta }}_{n}$ were unique and $\sqrt{n}({\hat{\theta }}_{n}-\theta )$ had an asymptotic distribution, one could use a Taylor series expansion for *g* to derive asymptotic results for ${\hat{\xi }}_{n}^{\mathrm{res}}$. Building on this idea, Condition 2.5 introduces a parametrization of a neighbourhood of $\xi_{0}$ that allows applying standard tools in order to obtain the asymptotic distribution of the restricted M-estimator.

Condition 2.5 Given $\xi_{0}\in g(\Theta ),$ there exists an open set M in $\Xi^{\mathrm{res}}$ with $\xi_{0}\in M\subset g(\Theta ),$ and (S,h), where S is an open set in $R^{q_{s}},$$q_{s}\leq q,$ and h:S→M is one-to-one, bi-continuous and twice continuously differentiable, with $\xi_{0}=h(s_{0})$ for some $s_{0}\in S$.

Under the setting in Condition 2.5, we will prove that ${\hat{s}}_{n}=h^{-1}({\hat{\xi }}_{n}^{\mathrm{res}})$ is a strong M-estimator for the criterion function $P_{n}m_{h(s)}$ over S. Then, we can apply Theorem 5.23 of van der Vaart [14] to obtain the asymptotic behaviour of ${\hat{s}}_{n}$, which, combined with a Taylor expansion of *h* about $s_{0}$, yield a linear expansion for ${\hat{\xi }}_{n}^{\mathrm{res}}$. Finally, requiring Condition 2.6, which relates the parametrizations (S,h) and (Θ,g), suffices to derive the asymptotic distribution of ${\hat{\xi }}_{n}^{\mathrm{res}}$ in terms of *g*.

Condition 2.6 Consider (Θ,g) as in Condition 2.2 and (S,h) as in Condition 2.5. For each $\theta_{0}\in g^{-1}(\xi_{0}),$$\mathrm{span}\nabla g(\theta_{0})=\mathrm{span}\nabla h(s_{0})$.

Condition 2.6 ensures that $T_{\xi_{0}}=\mathrm{span}\nabla g(\theta_{0})$ is well defined regardless of the fact that $g^{-1}(\xi_{0})$ may contain multiple $\theta_{0}$'s. Moreover, $T_{\xi_{0}}$ also agrees with $\mathrm{span}\nabla h(s_{0})$. Consequently, the orthogonal projection $\Pi_{\xi_{0}(\Sigma )}$ onto $T_{\xi_{0}}$ with respect to the inner product defined by a symmetric positive definite matrix Σ satisfies

(6) $$\begin{array}{rl} \Pi_{\xi_{0}(\Sigma )} & =\nabla g(\theta_{0})(\nabla g(\theta_{0}{)}^{T}\Sigma \nabla g(\theta_{0}){)}^{†}\nabla g(\theta_{0}{)}^{T}\Sigma \\ & =\nabla h(s_{0})(\nabla h(s_{0}{)}^{T}\Sigma \nabla h(s_{0}){)}^{-1}\nabla h(s_{0}{)}^{T}\Sigma , \end{array}$$

where $A^{†}$ denotes a generalized inverse of the matrix *A*. The gradient of *g* is not necessarily of full rank, in contrast to the gradient of *h*. Note that $\Pi_{\xi_{0}(\Sigma )}$ is idempotent ($\Pi_{\xi_{0}(\Sigma )}^{2}=\Pi_{\xi_{0}(\Sigma )}$) and the span of its columns is equal to $T_{\xi_{0}}$. However, $\Pi_{\xi_{0}(\Sigma )}$ is not in general symmetric, but rather self-adjoint with respect to the inner product induced by Σ since $<x,y{>}_{\Sigma }:=y^{T}\Sigma x$.

Proposition 2.7 Assume that Conditions 2.2, 2.5 and 2.6 hold. Assume also that the unrestricted problem satisfies the regularity Conditions A.2–A.4 in the Appendix. Then, any strong M-estimator ${\hat{\xi }}_{n}^{\mathrm{res}}$ of the restricted problem that converges in probability to $\xi_{0}$ satisfies

(7) $$\sqrt{n}({\hat{\xi }}_{n}^{\mathrm{res}}-\xi_{0})=\frac{1}{\sqrt{n}}\sum_{i=1}^{n}\Pi_{\xi_{0}(-V_{\xi_{0}})}IF_{\xi_{0}}(Z_{i})+o_{p}(1),$$

where $IF_{\xi_{0}}(Z_{i})=-V_{\xi_{0}}^{-1}{\dot{m}}_{\xi_{0}}(Z_{i})$ is the influence function of the unrestricted estimator defined in Equation (5), $V_{\xi_{0}}$ is the non-singular symmetric second derivative matrix of M(ξ) at $\xi_{0},$ and $\Pi_{\xi_{0}(-V_{\xi_{0}})}$ is defined according to Equation (6).

Moreover, $\sqrt{n}({\hat{\xi }}_{n}^{\mathrm{res}}-\xi_{0})$ is asymptotically normal with mean zero and asymptotic variance

(8) $$\text{avar}\{\sqrt{n}({\hat{\xi }}_{n}^{\mathrm{res}}-\xi_{0})\}=\Pi_{\xi_{0}(-V_{\xi_{0}})}V_{\xi_{0}}^{-1}P\left\{{\dot{m}}_{\xi_{0}}{\dot{m}}_{\xi_{0}}^{T}\right\}V_{\xi_{0}}^{-1}\Pi_{\xi_{0}(-V_{\xi_{0}})}^{T}.$$

As an aside remark, we conjecture that for estimators that are maximizers of a criterion function under restrictions that satisfy Conditions 2.2–2.6, when Equation (5) is true, Equation (7) also holds. This can be important since the asymptotic distribution of restricted estimators will be derived directly from the asymptotic distribution of the unrestricted one.

The optimization problem that defines the restricted M-estimators considered in this paper is, in general, not convex and hence difficult to solve. However, in the case of maximum likelihood estimation in reduced rank multivariate generalized linear models, the main application of the results in our paper, efficient algorithms exist for solving it (see [6] and the VGAMR package).

## 3. Asymptotic theory for the maximum likelihood estimator in reduced rank multivariate generalized linear models

In this section we show that maximum likelihood estimators in reduced-rank multivariate generalized linear models are restricted strong M-estimators for the conditional log-likelihood. Using results in Section 2.1, we obtain the existence, consistency and asymptotic distribution of maximum likelihood estimators in reduced-rank multivariate generalized linear models. In practice, these estimators can be obtained using the R package VGAM, developed by Yee [15].

### 3.1. Exponential family

Let $Y=(Y_{1},\ldots ,Y_{q}{)}^{T}$ be a *q*-dimensional random vector and assume that its distribution belongs to a *k*-parameter canonical exponential family with pdf (pms)

(9) $$f_{\eta }(y)=\exp\{\eta^{T}T(y)-\psi (\eta )\}h(y),$$

where $T(y)=(T_{1}(y),\ldots ,T_{k}(y){)}^{T}$ is a vector of known real-valued functions, h(y)≥0 is a non-negative known function and $\eta \in R^{k}$ is the vector of natural parameters, taking values in

(10) $$H=\{\eta \in R^{k}:\int \exp\{\eta^{T}T(y)\}h(y)\;dy<\infty \},$$

where the integral is replaced by a sum when *Y* is discrete. The set *H* of the natural parameter space is assumed to be open and convex in $R^{k}$, and *ψ* a strictly convex function defined on *H*. Moreover, we assume ψ(η) is convex and infinitely differentiable in *H*. In particular,

(11) $$\nabla \psi (\eta )=E_{\eta }^{T}(T(Y))\;\text{and}\;\nabla^{2}\psi (\eta )={\mathrm{var}}_{\eta }(T(Y)),\;\text{for every}\eta \in H,$$

where $\nabla^{2}\psi$ is the k×k matrix of second derivatives of *ψ*. Since *ψ* is strictly convex, ${\mathrm{var}}_{\eta }(T(Y))$ is non-singular for every η∈H.

### 3.2. Multivariate generalized linear models

Let Z=(X,Y) be a random vector, where now $Y\in R^{q}$ is a multivariate response and $X\in R^{p}$ is a vector of predictors. The multivariate generalized linear model postulates that the conditional distribution of *Y* given *X* belongs to some fixed exponential family and hypothesizes that the *k*-vector of natural parameters, which we henceforth call $\eta_{x}$ to emphasize the dependence on *x*, depends linearly on the vector of predictors. Thus, the pdf (pms) of Y∣X=x is given by

(12) $$f_{Y|X=x}(y)=\exp\{\eta_{x}^{T}T(y)-\psi (\eta_{x})\}h(y),$$

where $\eta_{x}\in R^{k}$ depends linearly on *x*.

Frequently, a subset of the natural parameters depends on *x*, whereas its complement does not. The normal linear model with constant variance is such an example. To accommodate this structure, we partition the vector $\eta_{x}$ indexing model (12) into $\eta_{x1}$ and $\eta_{x2}$, with $k_{1}$ and $k_{2}$ components, and assume that *H*, the natural parameter space of the exponential family, is $R^{k_{1}}\times H_{2}$, where $H_{2}$ is an open convex subset of $R^{k_{2}}$. We also assume that

(13) $$\eta_{x}=\left(\begin{array}{c} \eta_{x1}\\ \eta_{x2} \end{array}\right)=\left(\begin{array}{c} {\bar{\eta }}_{1}+\beta x\\ {\bar{\eta }}_{2} \end{array}\right),$$

where $\beta \in R^{k_{1}\times p}$, ${\bar{\eta }}_{1}\in R^{k_{1}}$ and ${\bar{\eta }}_{2}\in H_{2}$. Let $\xi =({\bar{\eta }}_{1}^{T},{\mathrm{vec}}^{T}(\beta ),{\bar{\eta }}_{2}^{T}{)}^{T}\in \Xi =R^{k_{1}}\times R^{k_{1}p}\times H_{2}$, denote a generic vector and $\xi_{0}$ the true parameter. Suppose *n* independent and identically distributed copies of Z=(X,Y) satisfying Equations (12) and (13), with true parameter vector $\xi_{0}$, are available. Given a realization $z_{i}=(x_{i},y_{i})$, i=1,…,n, the conditional log-likelihood, up to a factor that does not depend on the parameter of interest, is

(14) $$L_{n}(\beta ;\bar{\eta })=\frac{1}{n}\sum_{i=1}^{n}\left\{\eta_{x_{i}}^{T}T(y_{i})-\psi (\eta_{x_{i}})\right\}.$$

Let

(15) $$m_{\left(\beta ;\bar{\eta }\right)}(z)=\eta_{x}^{T}T(y)-\psi (\eta_{x}).$$

By definition (3), the maximum likelihood estimator (MLE) of the parameter indexing model (12) subject to (13), if it exists, is an M-estimator.

Theorem 3.1 next establishes the existence, consistency and asymptotic normality of ${\hat{\xi }}_{n}$, the MLE of $\xi_{0}$.

Theorem 3.1 Assume that Z=(X,Y) satisfies model (12) subject to (13) with true parameter $\xi_{0}$. Under regularity conditions (A20), (A21), (A22) and (A23) in the Appendix, the maximum likelihood estimate of $\xi_{0},$${\hat{\xi }}_{n},$ exists, is unique and converges in probability to $\xi_{0}$. Moreover, $\sqrt{n}({\hat{\xi }}_{n}-\xi_{0})$ is asymptotically normal with covariance matrix

(16) $$W_{\xi_{0}}={\left[E\left\{F(X{)}^{T}\nabla^{2}\psi (F(X)\xi_{0})F(X)\right\}\right]}^{-1},$$

where

$$F(x)=\left(\begin{array}{cc} \left(\left(1,x^{T}\right)⊗I_{k_{1}}\right) & 0\\ 0 & I_{k_{2}} \end{array}\right),$$

and $\nabla^{2}\psi$ was defined in Equation (11).

### 3.3. Partial reduced rank multivariate generalized linear models

When the number of natural parameters or the number of predictors is large, the precision of the estimation and/or the interpretation of results can be adversely affected. A way to address this is to assume that the parameters live in a lower dimensional space. That is, we assume that the vector of predictors can be partitioned as $x=(x_{1}^{T},x_{2}^{T}{)}^{T}$ with $x_{1}\in R^{r}$ and $x_{2}\in R^{p-r}$, and that the parameter corresponding to $x_{1}$, $\beta_{1}\in R^{k_{1}\times r}$, has rank $d<\min\{k_{1},r\}$. In this way, the natural parameters $\eta_{x}$ in Equation (12) are related to the predictors via

(17) $$\eta_{x}=\left(\begin{array}{c} \eta_{x1}\\ \eta_{x2} \end{array}\right)=\left(\begin{array}{c} {\bar{\eta }}_{1}+\beta_{1}x_{1}+\beta_{2}x_{2}\\ {\bar{\eta }}_{2} \end{array}\right),$$

where $\beta_{1}\in R_{d}^{k_{1}\times r}$, the set of matrices in $R^{k_{1}\times r}$ of rank $d\leq \min\{k_{1},r\}$, while $\beta_{2}\in R^{k_{1}\times (p-r)}$, and $\beta =(\beta_{1},\beta_{2})$.

Following Yee and Hastie [6], we refer to the exponential conditional model (12) subject to the restrictions imposed in Equation (17) as *partial reduced rank multivariate generalized linear model*. The reduced-rank multivariate generalized linear model is a special case of model (17) with $\beta_{2}=0$.

To obtain the asymptotic distribution of the M-estimators for this reduced model, we will show that Conditions 2.2–2.6 are satisfied for $\Xi^{\mathrm{res}}$, (Θ,g), M and (S,h), which are defined next. To maintain consistency with notation introduced in Section 2.1, we vectorize each matrix involved in the parametrization of our model and reformulate the parameter space accordingly for each vectorized object. With this understanding, we use the symbol ≅ to indicate that a matrix space component in a product space is identified with its image through the operator $\mathrm{vec}:R^{m\times n}\to R^{mn}$. In the sequel, to keep the notation as simple as possible, we concatenate column vectors without transposing them; that is, we write (a,b) for $(a^{T},b^{T}{)}^{T}$. Moreover, we write $\xi =({\bar{\eta }}_{1},\beta ,{\bar{\eta }}_{2})$, with the understanding that *β* stands for vec(β).

For the non-restricted problem, $\beta_{1}$ belongs to $R^{k_{1}\times r}$, so that the entire parameter $\xi =({\bar{\eta }}_{1},\beta_{1},\beta_{2},{\bar{\eta }}_{2})$ belongs to

(18) $$\Xi ≅R^{k_{1}}\times R^{k_{1}\times r}\times R^{k_{1}\times (p-r)}\times H_{2}.$$

However, for the restricted problem, we assume that the true parameter $\xi_{0}=({\bar{\eta }}_{01},\beta_{01},\beta_{02},{\bar{\eta }}_{02})$ belongs to

(19) $$\Xi^{\mathrm{res}}≅R^{k_{1}}\times R_{d}^{k_{1}\times r}\times R^{k_{1}\times (p-r)}\times H_{2}.$$

Let

(20) $$\Theta ≅R^{k_{1}}\times \left\{R_{d}^{k_{1}\times d}\times R_{d}^{d\times r}\right\}\times R^{k_{1}\times (p-r)}\times H_{2}$$

and consider $g:\Theta \to \Xi^{\mathrm{res}}$, with $\left({\bar{\eta }}_{1},(S,T),\beta_{2},{\bar{\eta }}_{2})\mapsto ({\bar{\eta }}_{1},ST,\beta_{2},{\bar{\eta }}_{2}\right)$. Without loss of generality, we assume that $\beta_{01}\in R_{\mathrm{first},d}^{k_{1}\times r}$, the set of matrices in $R_{d}^{k_{1}\times r}$ whose first *d* rows are linearly independent. Therefore,

(21) $$\beta_{01}=\left(\begin{array}{c} I_{d}\\ A_{0} \end{array}\right)B_{0},\;with\;A_{0}\in R^{(k_{1}-d)\times d}\;and\;B_{0}\in R_{d}^{d\times r}.$$

This is trivial since $\beta_{01}\in R_{d}^{k_{1}\times r}$ and its first *d* rows are linearly independent. Consider

(22) $$M≅R^{k_{1}}\times R_{\mathrm{first},d}^{k_{1}\times r}\times R^{k_{1}\times (p-r)}\times H_{2},\;\text{and so}\;\xi_{0}\in M\subset \Xi^{\mathrm{res}}.$$

Finally, let

(23) $$S≅R^{k_{1}}\times \left\{R^{(k_{1}-d)\times d}\times R_{d}^{d\times r}\right\}\times R^{k_{1}\times (p-r)}\times H_{2},$$

and h:S→M be the map

(24) $$\left({\bar{\eta }}_{1},(A,B),\beta_{2},{\bar{\eta }}_{2}\right)\;\mapsto \;\left({\bar{\eta }}_{1},\left(\begin{array}{c} B\\ AB \end{array}\right),\beta_{2},{\bar{\eta }}_{2}\right).$$

Proposition 3.2 Conditions 2.2–2.6 are satisfied for $\xi_{0},$ Ξ, $\Xi^{\mathrm{res}},$(Θ,g),M and (S,h) defined in Equations (18)–(24), respectively.

Under the rank restriction on $\beta_{1}$ in Equation (17), the existence of the maximum likelihood estimator cannot be guaranteed in the sense of an M-estimator as defined in Equation (3) with $m_{\xi }=m_{\left(\beta ;\bar{\eta }\right)}$ in Equation (15), and Ξ replaced by $\Xi^{\mathrm{res}}$ in Equation (18). However, we can work with a strong M-estimator sequence for the criterion function $P_{n}m_{\left(\beta ;\bar{\eta }\right)}$ over $\Xi^{\mathrm{res}}$ using Lemma 2.3. Theorem 3.3 states our main contribution.

Theorem 3.3 Let $\xi_{0}=({\bar{\eta }}_{01},\beta_{01},\beta_{02},{\bar{\eta }}_{02})$ denote the true parameter value of $\xi =({\bar{\eta }}_{1},\beta_{1},\beta_{2},{\bar{\eta }}_{2})$. Assume that Z=(X,Y) satisfies model (12) subject to (17), with $\xi_{0}\in \Xi^{\mathrm{res}}$ defined in Equation (19). Then, there exists a strong maximizing sequence for the criterion function $P_{n}m_{\xi }$ over $\Xi^{\mathrm{res}}$ for $m_{\xi }=m_{\left(\beta ;\bar{\eta }\right)}$ defined in Equation (15). Moreover, any weak M-estimator sequence $\left\{{\hat{\xi }}_{n}^{\mathrm{res}}\right\}$ converges to $\xi_{0}$ in probability.

If $\left\{{\hat{\xi }}_{n}^{\mathrm{res}}\right\}$ is a strong M-estimator sequence, then $\sqrt{n}({\hat{\xi }}_{n}^{\mathrm{res}}-\xi_{0})$ is asymptotically normal with covariance matrix

(25) $$\begin{array}{rl} \text{avar}\{\sqrt{n}({\hat{\xi }}_{n}^{\mathrm{res}}-\xi_{0})\} & =\Pi_{\xi_{0}(W_{\xi_{0}}^{-1})}W_{\xi_{0}}\\ & =G(G^{T}W_{\xi_{0}}^{-1}G{)}^{-1}G^{T}, \end{array}$$

where $W_{\xi_{0}}$ is defined in Equation (16) and

(26) $$G=\left(\begin{array}{ccccc} I_{k_{1}} & 0 & 0 & 0 & 0\\ 0 & B_{0}^{T}⊗I_{k_{1}} & I_{r}⊗C_{0} & 0 & 0\\ 0 & 0 & 0 & I_{k_{1}(p-r)} & 0\\ 0 & 0 & 0 & 0 & I_{k_{2}} \end{array}\right),$$

with $\beta_{01}=C_{0}B_{0},$$C_{0}\in R^{k_{1}\times d}$ and $B_{0}\in R^{d\times r}$ any decomposition of $\beta_{01}$.

Remark 3.4 The asymptotic variance of the estimator does not depend on the specific decomposition of $\beta_{01}$. That is, for another $\beta_{01}={\tilde{C}}_{0}{\tilde{B}}_{0}$, even if (26) changes, (25) remains the same.

Remark 3.5 A plug-in procedure can be used to estimate the elements of the matrix in Equation  (25) and then, appealing to Slutzky's Theorem, asymptotic confidence regions for ${\hat{\xi }}_{n}^{\mathrm{res}}$ can be constructed. Also, bootstrap variance estimates can be computed by sampling with replacement from the empirical distribution, leading to so-called normal bootstrap intervals (see, e.g. [16], Section 8.3).

Remark 3.6 Since $\Pi_{\xi_{0}(W_{\xi_{0}}^{-1})}$ is a projection, $\Pi_{\xi_{0}(W_{\xi_{0}}^{-1})}W_{\xi_{0}}\leq W_{\xi_{0}}$. That is, the eigenvalues of $W_{\xi_{0}}-\Pi_{\xi_{0}(W_{\xi_{0}}^{-1})}W_{\xi_{0}}$ are non-negative, so that using partial reduced-rank multivariate generalized linear models results in efficiency gain.

Remark 3.7 Decomposing *x* into more sets of predictors with reduced rank parameters amounts to adding new rows, like the second set of rows of *G* in Equation (26), for each reduced rank parameter.

Remark 3.8 For more general families, like the one considered by Yee and Hastie [6], if the family in question satisfies the regularity conditions in Proposition 2.7, the asymptotic variance of the estimators can also be computed by making the corresponding substitutions in the formulas of Proposition 2.7.

## 4. Application: marital status in a workforce study

Yee and Hastie [6] analyse data from a self-administered questionnaire collected in a large New Zealand workforce observational study conducted during 1992–1993. For homogeneity, the analysis was restricted to a subset of 4105 European males with no missing values in any of the variables used. Yee and Hastie [6] were interested in exploring whether certain lifestyle and psychological variables were associated with marital status, especially separation/divorce. The response variable is Y=maritalstatus, with levels 1=single, 2=separatedordivorced, 3=widower, and 4=marriedorlivingwithapartner. The married/partnered are the reference group. Data on 14 regressors were collected, 12 of which are binary (1/0 for presence/absence, respectively). These have been coded so that their presence is negative healthwise. Their goal was to investigate if and how these 12 *unhealthy* variables were related to *Y* , adjusting for age and level of education. The variables are described in Table 1.

**Table 1.** Variables used in the workforce study.

Variable name	Description
marital	Marital status. 1=single, 2=separated or divorced, 3=widower, and 4=married or living with a partner
age30	Age −30, in years
logedu1	log⁡(1+ years of education at secondary or high school)
binge	In the last 3 months what is the largest number of drinks that you had on any one day? (1=20 or more, 0=less than 20)
smokenow	Current smoker?
sun	Does not usually wear a hat, shirt or suntan lotion when outside during summer
nerves	Do you suffer from ‘nerves’?
nervous	Would you call yourself a ‘nervous’ person?
hurt	Are your feelings easily hurt?
tense	Would you call yourself tense or ‘highly strung’?
miserable	Do you feel ‘just miserable’ for no reason?
fedup	Do you often feel ‘fed-up’?
worry	Do you worry about awful things that might happen?
worrier	Are you a worrier?
mood	Does your mood often go up and down?

A categorical response *Y* taking values 1,2,3,4 with probabilities $\mathrm{pr}(Y=i)=p_{i}$ can be expressed as a multinomial vector to fit the generalized linear model presented in this paper, $Y=(Y_{1},Y_{2},Y_{3},Y_{4}{)}^{T}$, where $Y_{i}=1$ if *Y* =*i* and $Y_{i}=0$ otherwise, and $\sum_{i=1}^{4}Y_{i}=1$. The pdf of *Y* can be written as

(27) $$f_{Y}(y)=\exp\{\eta^{T}T(y)-\psi (\eta )\}h(y),$$

where $y=(y_{1},y_{2},y_{3},y_{4})$, $T(y)=(y_{1},y_{2},y_{3})$, $\eta =(\eta_{1},\eta_{2},\eta_{3})\in R^{3}$, $\psi (\eta )=1+\sum_{i=1}^{3}\exp(\eta_{i})$ and $h(y)=1/(y_{1}!y_{2}!y_{3}!(1-y_{1}-y_{2}-y_{3})!)$. The natural parameter $\eta =(\eta_{1},\eta_{2},\eta_{3})\in R^{3}$, is related to the pdf of *Y* through the identity $\eta_{i}=\log(p_{i}/p_{4})$, *i*=1,2,3.

Let *X* be the vector of predictor variables and consider $p_{i}=p_{i}(x)=\mathrm{pr}(Y_{i}=1|X=x)$. The dependence of $p_{i}$ on *x* will not be made explicit in the notation. As in [6] we fit a multinomial regression model to Y|X=x, as in Equation (27), with

(28) $$\eta_{x}=\left(\begin{array}{c} \log(p_{1}/p_{4})\\ \log(p_{2}/p_{4})\\ \log(p_{3}/p_{4}) \end{array}\right)=\bar{\eta }+\beta x,$$

where $\bar{\eta }\in R^{3}$ is the intercept and $\beta \in R^{3\times 14}$ is the coefficient matrix, so that there are 3×14+3=42+3=45 parameters to estimate. When a multinomial linear model is fitted to the data at level 0.05, age30 and binge are significant for $\log(p_{1}/p_{4})$, smokehow and tense for $\log(p_{2}/p_{4})$, and only age30 is significant for $\log(p_{3}/p_{4})$.

We next fitted a partial reduced rank multivariate generalized linear model, where the two continuous variables, age30 and logedu1, were not subject to restriction. That is,

(29) $$\eta_{x}=\bar{\eta }+\beta x=\bar{\eta }+\beta_{1}x_{1}+\beta_{2}x_{2},$$

where $x_{2}$ represent the continuous variables and $x_{1}$ the 12 binary predictors. The AIC criterion estimates the rank of $\beta_{1}$ in Equation (29) to be one (see [6]). Using the asymptotic results from the current paper, Duarte [17] developed a test based on Bura and Yang [18] that also estimates the dimension to be 1. Therefore, in our notation, *q*=4, $k=k_{1}=3$, *p*=14, *r*=2, *d*=1, and $\beta_{1}=AB$, A:3×1 y B:1×12, $\beta_{2}:3\times 2$ and $\bar{\eta }:3\times 1$. The rank restriction results in a drastic reduction in the total number of parameters from 45 to 24.

The reduction in the estimation burden is also reflected in how tight the confidence intervals are compared with those in the unrestricted model, as can be seen in Table 3 and Table 2 in [6]. As a consequence the variables nervous, hurt, which are not significant in the unrestricted generalized linear model, are significant in the reduced (29). Furthermore, some variables, such as binge, smokenow, nervous and tense, are now significant for all responses.

**Table 2.** Estimators with standard errors for $\beta_{1}$ for the generalized linear model (first two rows) and the reduced generalized linear model (last two rows).

Variable		$\log(p_{1}/p_{4})$	$\log(p_{2}/p_{4})$	$\log(p_{3}/p_{4})$
Intercept				
	GLM	−1.573*	−2.921*	−6.123*
		(0.388)	(0.396)	(1.106)
	PRR–GLM	−1.762*	−2.699*	−6.711*
		(0.377)	(0.383)	(1.047)
age30				
	GLM	−0.190*	0.012	0.077*
		(0.008)	(0.008)	(0.024)
	PRR–GLM	−0.191*	0.012	0.086*
		(0.008)	(0.008)	(0.023)
logedu1				
	GLM	0.254	−0.316	−0.198
		(0.228)	(0.214)	(0.566)
	PRR–GLM	0.338	−0.365	−0.089
		(0.225)	(0.213)	(0.559)

${}^{*}$ Significance at 5% level.

**Table 3.** MLEs with their standard errors in parentheses for the full rank generalized linear model (first two rows) and the partial reduced rank generalized linear model (last two rows).

Variable		$\log(p_{1}/p_{4})$	$\log(p_{2}/p_{4})$	$\log(p_{3}/p_{4})$
binge				
	GLM	0.801*	0.318	1.127
		(0.143)	(0.256)	(0.670)
	PRR–GLM	0.569*	0.786*	1.114*
		(0.125)	(0.196)	(0.409)
smokenow				
	GLM	0.022	0.501*	0.654
		(0.126)	(0.157)	(0.469)
	PRR–GLM	0.222*	0.306*	0.434*
		(0.088)	(0.119)	(0.208)
sun				
	GLM	−0.066	0.120	−0.088
		(0.122)	(0.161)	(0.518)
	PRR–GLM	0.011	0.015	0.021
		(0.084)	(0.116)	(0.164)
nerves				
	GLM	−0.102	0.123	−1.456
		(0.138)	(0.198)	(0.841)
	PRR–GLM	−0.054	−0.074	−0.105
		(0.101)	(0.139)	(0.197)
nervous				
	GLM	0.297	0.353	1.007
		(0.169)	(0.228)	(0.665)
	PRR–GLM	0.312*	0.430*	0.609*
		(0.124)	(0.168)	(0.291)
hurt				
	GLM	0.184	0.210	0.483
		(0.126)	(0.167)	(0.501)
	PRR–GLM	0.180*	0.248*	0.352
		(0.089)	(0.122)	(0.199)
tense				
	GLM	0.166	0.483*	1.108
		(0.176)	(0.214)	(0.612)
	PRR–GLM	0.302*	0.416*	0.590*
		(0.122)	(0.163)	(0.284)
miserable				
	GLM	−0.050	0.128	−0.093
		(0.138)	(0.178)	(0.613)
	PRR–GLM	0.019	0.0268	0.038
		(0.094)	(0.129)	(0.185)
fedup				
	GLM	0.112	0.249	−0.214
		(0.122)	(0.171)	(0.548)
	PRR–GLM	0.117	0.161	0.229
		(0.094)	(0.129)	(0.185)
worry				
	GLM	0.113	−0.102	−0.548
		(0.145)	(0.209)	(0.818)
	PRR–GLM	0.003	0.004	0.005
		(0.106)	(0.146)	(0.207)
worrier				
	GLM	−0.027	-0.243	−0.548
		(0.131)	(0.180)	(0.550)
	PRR–GLM	−0.116	-0.160	−0.227
		(0.092)	(0.128)	(0.193)
mood				
	GLM	−0.111	0.092	−0.193
		(0.123)	(0.171)	(0.553)
	PRR–GLM	−0.037	−0.052	−0.073
		(0.087)	(0.120)	(0.172)

${}^{*}$ Significant at 5% level.

All significant coefficients are positive. These correspond to the variables binge, smokenow, nervous, tense and hurt for single and divorced/separated groups. Since the positive value of the binary variables indicates poor lifestyle and negative psychological characteristics, our analysis concludes that for men with these features, the chance of being single, divorced or widowed is higher than the chance of being married, adjusting for age and education. Also, the coefficients corresponding to the response $\log(p_{3}/p_{4})$ are twice as large as those of $\log(p_{1}/p_{4})$, suggesting the effect of the predictors differs in each group. All computations were performed using the R package VGAM, developed by Yee [15]. The R script to reproduce the data analysis in Section 4 can be accessed at supplemental data.

## 5. Discussion

With the exception of the work of Yee and his collaborators on VGLMs [6,7,19], where the distribution of the response can be any member of the multivariate exponential family, reduced-rank regression has been almost exclusively restricted to regressions with continuous response variables. Estimation methods and the corresponding software for general partial reduced rank multivariate generalized linear models were developed by Yee and Hastie [6] and Yee [7]. Yet, the distribution and statistical properties of the estimators were not obtained. In this paper we fill this gap by developing asymptotic theory for the restricted rank maximum likelihood estimates of the parameter matrix in multivariate GLMs.

To illustrate the potential impact of our results, we refer to the real data analysis example in Section 4. In order to assess the significance of the predictors, Yee and Hastie [6] calculate the standard errors for the coefficient matrix factors, *A* and *B*, independently and can only infer about the significance of the components of the matrix *A* and the components of the matrix *B* separately. The asymptotic distribution for either estimator is obtained assuming that the other is fixed and known. In this way, they first analyse $\nu =Bx_{1}$ to check which predictors are significant and then Aν to examine how they influence each response. Their standard errors are ad-hoc and it is unclear what the product of standard errors measures as relates to the significance of the product of the components of the coefficient matrix $\beta_{1}=AB$. Moreover, this practical ad-hoc approach cannot readily be extended when *d*>1.

Using the results of Theorem 3.3, we can obtain the errors of each component of the coefficient matrices *A*, *B* simultaneously, and assess the statistical significance of each predictor on each response. Using the ad-hoc approach of Yee and Hastie [6], a predictor can only be found to be significant across all responses. For example, Yee and Hastie [6] find the predictor hurt to be significant for all three groups (single, divorced/separated, widower). On the other hand, we can assess the significance of any response/predictor combination. Thus, we find hurt to be significant for single and divorced/separated groups, but not for widower men group (see Table 3).

A potential future direction for our approach was brought to our attention by a referee. The computational cost for fitting a reduced rank multinomial logistic regression can be very high. Powers et al. [20] proposed replacing the rank restriction with a restriction on the nuclear norm which amounts to a convex relaxation of the reduced-rank multinomial regression problem. Our methodology can be adapted to obtain asymptotic inference for the regularized parameter estimates.

## Appendix. Proofs

The consistency of M-estimators has long been established (see, for instance, Theorem 5.7 in [14]). The functions M(ξ) and $M_{n}(\xi )$ are defined in Equation (2). Typically, the proof for the consistency of M-estimators assumes that $\xi_{0}$, the parameter of interest, is a *well-separated* point of maximum of *M*, which is ascertained by assumptions (a) and (b) of Proposition A.1. Assumption (c) of Proposition A.1 yields uniform consistency of $M_{n}$ as an estimator of *M*, a property needed in order to establish the consistency of M-estimators.

Proposition A.1 Assume (a) $m_{\xi }(z)$ is a strictly concave function in ξ∈Ξ, where Ξ is a convex open subset of a Euclidean space; (b) the function M(ξ) is well defined for any ξ∈Ξ and has a unique maximizer $\xi_{0};$ that is, $M(\xi_{0})>M(\xi )$ for any $\xi \neq \xi_{0};$ and (c) for any compact set K in Ξ,

(A1) $$E\left[\underset{\xi \in K}{\sup}\left|m_{\xi }(Z)\right|\right]<\infty .$$

Then, for each n∈N, there exists a unique M-estimator ${\hat{\xi }}_{n}$ for the criterion function $M_{n}$ over Ξ. Moreover, ${\hat{\xi }}_{n}\to \xi_{0}$ a.e. as n→∞.

Proof. For each compact subset K of Ξ, $\left\{m_{\xi }:\xi \in K\right\}$ is a collection of measurable functions which, by assumption (c), has an integrable envelope. Moreover, for each fixed *z*, the map $\xi \mapsto m_{\xi }(z)$ is continuous, since it is concave and defined on the open set Ξ. As stated in Example 19.8 of van der Vaart [14], these conditions guarantee that the class is Glivenko–Cantelli. That is,

(A2) $$\text{pr}\left(\lim_{n\to \infty }\underset{\xi \in K}{\sup}|P_{n}m_{\xi }-Pm_{\xi }|=0\right)=1.$$

We need to prove that there exists a unique maximizer of $M_{n}(\xi )=P_{n}\xi ,$ and that it converges to the maximizer of $M(\xi )=Pm_{\xi }$. We first consider the deterministic case ignoring for the moment that $\left\{M_{n}\right\}$ is a sequence of random functions. Since $\xi_{0}$ belongs to the open set Ξ, there exists $\epsilon_{0}>0$ such that the closed ball $B[\xi_{0},\epsilon_{0}]$ is contained in Ξ. Uniform convergence of $\left\{M_{n}\right\}$ to *M* over $K=\{\xi :∥\xi -\xi_{0}∥=\epsilon_{0}\}$ guarantees that

(A3) $$\lim_{n\to \infty }\underset{∥\xi -\xi_{0}∥=\epsilon_{0}}{\sup}\left\{M_{n}(\xi )-M_{n}(\xi_{0})\right\}=\underset{∥\xi -\xi_{0}∥=\epsilon_{0}}{\sup}\left\{M(\xi )-M(\xi_{0})\right\}<0$$

because $M(\xi_{0})>M(\xi )$ for any $\xi \neq \xi_{0}$. Then, for $n\geq n_{0}(\epsilon_{0})$,

(A4) $$\underset{∥\xi -\xi_{0}∥=\epsilon_{0}}{\sup}M_{n}(\xi )-M_{n}(\xi_{0})<0.$$

Let $\zeta_{n}(\xi )=M_{n}(\xi )-M_{n}(\xi_{0})$. Since $M_{n}$ is concave and continuous, $\zeta_{n}$ attains its maximum over the compact set $B[\xi_{0},\epsilon_{0}]$, which we denote by ${\hat{\xi }}_{n}$. Note that $\zeta_{n}(\xi_{0})=0$ and $\zeta_{n}$ is strictly smaller than zero in the boundary of the ball, as shown in Equation (A4); therefore, we conclude that ${\hat{\xi }}_{n}\in B(\xi_{0},\epsilon_{0})$, so that ${\hat{\xi }}_{n}$ is a local maximum for $\zeta_{n}$. Let *ξ* satisfy $∥\xi -\xi_{0}∥>\epsilon_{0}$. The convexity of Ξ implies there exists t∈(0,1) such that $\tilde{\xi }=(1-t)\xi_{0}+t\xi$ satisfies $∥\tilde{\xi }-\xi_{0}∥=\epsilon_{0}$, and therefore

(A5) $$\zeta_{n}({\hat{\xi }}_{n})\geq \zeta_{n}(\xi_{0})=0>\zeta_{n}(\tilde{\xi })\geq t\zeta_{n}(\xi )+(1-t)\zeta_{n}(\xi_{0})=t\zeta_{n}(\xi ),$$

implying that $\zeta_{n}(\xi )<0\leq \zeta_{n}({\hat{\xi }}_{n})$. Therefore, the maximum ${\hat{\xi }}_{n}\in B(\xi_{0},\epsilon_{0})$ is global. The strict concavity of $M_{n}$ guarantees that such global maximum is unique, thus ${\hat{\xi }}_{n}$ is the unique solution to the optimization problem in Equation (3). By repeating this argument for any $\epsilon <\epsilon_{0}$, we prove the convergence of the sequence $\left\{{\hat{\xi }}_{n}\right\}$ to $\xi_{0}$. Turning to the stochastic case, the uniform convergence of $M_{n}$ to *M* over $K=B[\xi_{0},\epsilon_{0}]$ on a set $\Omega_{1}$, with $\mathrm{pr}(\Omega_{1})=1$, as assumed in Equation (A2), guarantees the deterministic result can be applied to any element of $\Omega_{1}$, which obtains the result.

Proof of Lemma 2.3. Proof of Lemma 2.3 Let $\left\{{\hat{\xi }}_{n}\right\}$ be any (weak/strong) M-estimator sequence of the unconstrained maximization problem. Since $M_{n}({\hat{\xi }}_{n})\geq \underset{\xi \in \Xi }{\sup}M_{n}(\xi )-A_{n}$ with $A_{n}\to 0$, we have

(A6) $$1=\lim_{n\to \infty }\text{pr}\left(\underset{\xi \in \Xi }{\sup}P_{n}m_{\xi }<\infty \right)\leq \lim_{n\to \infty }\text{pr}\left(\underset{\xi \in \Xi^{\mathrm{res}}}{\sup}P_{n}m_{\xi }<\infty \right).$$

Define

(A7) $$\Omega_{n}:=\left\{\underset{\xi \in \Xi^{\mathrm{res}}}{\sup}P_{n}m_{\xi }<\infty \right\}.$$

For all *n*, there exists ${\hat{\xi }}_{n}^{\mathrm{res}}$ such that

(A8) $$M_{n}({\hat{\xi }}_{n}^{\mathrm{res}})\geq \underset{\xi \in \Xi^{\mathrm{res}}}{\sup}M_{n}(\xi )-\frac{1}{n^{2}}$$

on $\Omega_{n}$. Let ${\hat{\xi }}_{n}^{\mathrm{res}}=0$ on $\Omega_{n}^{c}$. Then, since $\text{pr}(\Omega_{n})\to 1$, $\left\{{\hat{\xi }}_{n}^{\mathrm{res}}\right\}$ is a strong M-estimator for the criterion function $M_{n}$ over $\Xi^{\mathrm{res}}$.

Proof of Proposition 2.4. Proof of Proposition 2.4 In Proposition A.1 we established the existence of a unique maximizer ${\hat{\xi }}_{n}$ for the criterion function $M_{n}$ over Ξ. We can now invoke Lemma 2.3 to guarantee the existence of ${\hat{\xi }}_{n}^{\mathrm{res}}$, a strong M-estimator for the criterion function $M_{n}$ over $\Xi^{\mathrm{res}}$. Let $\left\{{\hat{\xi }}_{n}^{\mathrm{res}}\right\}$ be any strong M-estimator for the criterion function $M_{n}$ over $\Xi^{\mathrm{res}}$. We start from the deterministic case:

(A9) $$M_{n}({\hat{\xi }}_{n}^{\mathrm{res}})\geq \underset{\xi \in \Xi^{\mathrm{res}}}{\sup}M_{n}(\xi )-A_{n},$$

where $M_{n}$ is defined in Equation (2) and $A_{n}$ is a sequence of real numbers with $A_{n}\to 0$. As in the proof of Proposition A.1, define $\zeta_{n}(\xi )=M_{n}(\xi )-M_{n}(\xi_{0})$ to obtain that, for $\epsilon_{0}$ small enough,

(A10) $$\underset{∥\xi -\xi_{0}∥=\epsilon_{0}}{\sup}\zeta_{n}(\xi )\leq \frac{1}{2}\underset{∥\xi -\xi_{0}∥=\epsilon_{0}}{\sup}\left\{M(\xi )-M(\xi_{0})\right\}:=-\delta (\epsilon_{0})$$

for *n* large enough. Under Condition 2.2, $\xi_{0}\in \Xi^{\mathrm{res}}$, and therefore, by Equation (A9),

(A11) $$\zeta_{n}({\hat{\xi }}_{n}^{\mathrm{res}})=M_{n}({\hat{\xi }}_{n}^{\mathrm{res}})-M_{n}(\xi_{0})\geq M_{n}({\hat{\xi }}_{n}^{\mathrm{res}})-\underset{\xi \in \Xi^{\mathrm{res}}}{\sup}M_{n}(\xi )\geq -A_{n}.$$

Since $A_{n}\to 0$, $-A_{n}>-\delta (\epsilon_{0})$ for *n* large enough. Combining this with Equations (A10) and (A11) obtains

$$\underset{∥\xi -\xi_{0}∥=\epsilon_{0}}{\sup}\zeta_{n}(\xi )<\zeta_{n}({\hat{\xi }}_{n}^{\mathrm{res}}).$$

We will deduce that $∥{\hat{\xi }}_{n}^{\mathrm{res}}-\xi_{0}∥<\epsilon_{0}$, once we prove that

(A12) $$\underset{∥\xi -\xi_{0}∥=\epsilon_{0}}{\sup}\zeta_{n}(\xi )=\underset{∥\xi -\xi_{0}∥\geq \epsilon_{0}}{\sup}\zeta_{n}(\xi ).$$

Now, let us prove Equation (A12). Choose *ξ* with $∥\xi -\xi_{0}∥>\epsilon_{0}$, and take t∈(0,1) such that $\tilde{\xi }=(1-t){\hat{\xi }}_{n}+t\xi$ is a distance $\epsilon_{0}$ from $\xi_{0}$, where ${\hat{\xi }}_{n}$ is the maximizer of $\zeta_{n}$ over Ξ, as defined in Proposition A.1, which is assumed to be at distance smaller than $\epsilon_{0}$ from $\xi_{0}$. Then,

$$\zeta_{n}(\tilde{\xi })=\zeta_{n}\left((1-t){\hat{\xi }}_{n}+t\xi \right)\geq (1-t)\zeta_{n}({\hat{\xi }}_{n})+t\zeta_{n}(\xi )\geq \zeta_{n}(\xi ).$$

Thus,

$$\underset{∥\xi -\xi_{0}∥=\epsilon_{0}}{\sup}\zeta_{n}(\xi )\geq \zeta_{n}(\tilde{\xi })\geq \zeta_{n}(\xi ),\;\text{for any }\xi \text{ with }∥\xi -\xi_{0}∥>\epsilon_{0},$$

which in turn yields (A12). When $A_{n}=o_{p}(1)$, convergence in probability of $\left\{{\hat{\xi }}_{n}^{\mathrm{res}}\right\}$ to $\xi_{0}$ is equivalent to the existence of an almost everywhere convergent sub-sub-sequence for any subsequence $\left\{{\hat{\xi }}_{n_{k}}^{\mathrm{res}}\right\}$. Therefore, by applying the deterministic result to the set of probability one, where there exists a sub-subsequence of $A_{n_{k}}$ that converges to zero a.e. we obtain the result.

Regularity conditions for Proposition 2.7 (from Theorem 5.23, p. 53 in [14]).

Condition A.2 For each *ξ* in an open subset Ξ of a Euclidean space, $m_{\xi }(z)$ is a measurable function in *z* such that $m_{\xi }(z)$ is differentiable in $\xi_{0}$ for almost every *z* with derivative ${\dot{m}}_{\xi_{0}}(z)$.

Condition A.3 There exists a measurable function ϕ(z) with $P\phi^{2}<\infty$ such that, for any $\xi_{1}$ and $\xi_{2}$ in a neighbourhood of $\xi_{0},$

(A13) $$|m_{\xi_{1}}(z)-m_{\xi_{2}}(z)|\leq \phi (z)∥\xi_{1}-\xi_{2}∥.$$

Condition A.4 The map $\xi \to Pm_{\xi }$ admits a second-order Taylor expansion at a point of maximum $\xi_{0}$ with non-singular symmetric second derivative matrix $V_{\xi_{0}}$.

Under regularity conditions A.2–A.4, van der Vaart proved in Theorem 5.23 of his book [14] that if $\left\{{\hat{\xi }}_{n}\right\}$ is a strong M-estimator sequence for the criterion function $P_{n}m_{\xi }$ over Ξ and ${\hat{\xi }}_{n}\to \xi_{0}$ in probability, then

(A14) $$\sqrt{n}({\hat{\xi }}_{n}-\xi_{0})=-V_{\xi_{0}}^{-1}\frac{1}{\sqrt{n}}\sum_{i=1}^{n}{\dot{m}}_{\xi_{0}}(Z_{i})+o_{p}(1).$$

Moreover, $\sqrt{n}({\hat{\xi }}_{n}-\xi_{0})$ is asymptotically normal with mean zero and

(A15) $$\text{avar}\left\{\sqrt{n}({\hat{\xi }}_{n}-\xi_{0})\right\}=V_{\xi_{0}}^{-1}P{\dot{m}}_{\xi_{0}}{\dot{m}}_{\xi_{0}}^{T}V_{\xi_{0}}^{-1}.$$

This result will be invoked in the following proofs.

Proof of Proposition 2.7. Proof of Proposition 2.7 Assume that $\left\{{\hat{\xi }}_{n}^{\mathrm{res}}\right\}$ is a sequence in $\Xi^{\mathrm{res}}$ that converges in probability to $\xi_{0}\in M$, which is assumed to be open in $\Xi^{\mathrm{res}}$. Then, $\mathrm{pr}({\hat{\xi }}_{n}^{\mathrm{res}}\in M)\to 1$. Bicontinuity of *h* guarantees that $s_{n}^{*}=h^{-1}({\hat{\xi }}_{n}^{\mathrm{res}})$ converges in probability to $s_{0}=h^{-1}(\xi_{0})$. Note that

(A16) $$P_{n}m_{h(s_{n}^{*})}=P_{n}m_{{\hat{\xi }}_{n}^{\mathrm{res}}}\geq \underset{\xi \in \Xi^{\mathrm{res}}}{\sup}P_{n}m_{\xi }\geq \underset{\xi \in M}{\sup}P_{n}m_{\xi }\geq \underset{s\in S}{\sup}P_{n}m_{h(s)},$$

except for an $o_{p}(n^{-1})$ term that is omitted in the last three inequalities. Therefore, $\left\{s_{n}^{*}\right\}$ is a strong maximizing sequence for the criterion function $P_{n}m_{h(s)}(z)$ over S. We next verify Conditions A.2–A.4 are satisfied for $\left\{s_{n}^{*}\right\}$, $s_{0}$, $m_{h(s)}(z)$ and $Pm_{h(s)}$. Specifically, Condition A.2 holds since $m_{h(s)}$ is a measurable function in *z* for all s∈S and $m_{h(s)}(z)$ is differentiable in $s_{0}$ for almost every *z*. In fact, $h(s_{0})=\xi_{0}$, $m_{\xi }(z)$ is differentiable at $\xi_{0}$ and h(s) is also differentiable. Moreover, the derivative function is $\nabla h(s_{0}){\dot{m}}_{\xi_{0}}$. For all $s_{1}$ and $s_{2}$ in a neighbourhood of $s_{0}$, by the continuity of *h*, $h(s_{1})$ and $h(s_{2})$ are in a neighbourhood of $\xi_{0}$. Then

$$\begin{array}{rl} |m_{h(s_{1})}(z)-m_{h(s_{2})}(z)| & \leq \phi (z)∥h(s_{1})-h(s_{2})∥\\ & \leq \phi (z)∥\nabla h{∥}_{\infty ,N_{s_{0}}}∥s_{1}-s_{2}∥, \end{array}$$

where $∥\nabla h{∥}_{\infty ,N_{s_{0}}}$ denotes the maximum of ∥∇h(s)∥ in a neighbourhood $N_{s_{0}}$ of $s_{0}$. The first inequality holds because such condition is valid in the unconstrained problem and the second inequality follows since *h* is continuously differentiable at $s_{0}$. Thus, the Lipschitz Condition A.3 is satisfied. For Condition A.4, we observe that the function $s\mapsto Pm_{h(s)}$ is twice continuously differentiable in $s_{0}$ because both $Pm_{\xi }$ and h(s) satisfy the required regularity properties at $\xi_{0}$ and $s_{0}$, respectively. Moreover, since $P{\dot{m}}_{\xi_{0}}=0$, the second derivative matrix of $Pm_{h(s)}$ at $s_{0}$, is $W_{s_{0}}=\nabla h(s_{0}{)}^{T}V_{\xi_{0}}\nabla h(s_{0})$, where $V_{\xi_{0}}$ is the second derivative matrix of $Pm_{\xi }$ at $\xi_{0}$. The matrix $W_{s_{0}}$ is non-singular and symmetric because $\nabla h(s_{0})$ is full rank and $V_{\xi_{0}}$ is non-singular and symmetric. We can now apply Theorem 5.23 in [14], and obtain

(A17) $$\sqrt{n}(s_{n}^{*}-s_{0})=-{\left(\nabla h(s_{0}{)}^{T}V_{\xi_{0}}\nabla h(s_{0})\right)}^{-1}\frac{1}{\sqrt{n}}\sum_{i=1}^{n}\nabla h(s_{0}{)}^{T}{\dot{m}}_{\xi_{0}}(Z_{i})+o_{p}(1),$$

so that the first-order Taylor series expansion of $h(s_{n}^{*})$ around $s_{0}$ is

$$\begin{array}{rl} \sqrt{n}(h(s_{n}^{*})-h(s_{0})) & =\nabla h(s_{0})\sqrt{n}(s_{n}^{*}-s_{0}))+o_{p}(1)\\ & =-\frac{1}{\sqrt{n}}\sum_{i=1}^{n}\nabla h(s_{0}){\left(\nabla h(s_{0}{)}^{T}V_{\xi_{0}}\nabla h(s_{0})\right)}^{-1}\nabla h(s_{0}{)}^{T}{\dot{m}}_{\xi_{0}}(Z_{i})+o_{p}(1)\\ & =\frac{1}{\sqrt{n}}\sum_{i=1}^{n}\Pi_{\xi_{0}(-V_{\xi_{0}})}IF_{\xi_{0}}(Z_{i})+o_{p}(1), \end{array}$$

for $\Pi_{\xi_{0}(-V_{\xi_{0}})}$ and $IF_{\xi_{0}}(z)$ defined in Equations (6) and (5), respectively, which obtains the expansion in Equation (7). Now, Equation (8) follows immediately.

Proof of Theorem 3.1. Proof of Theorem 3.1 Write

$$\eta_{x1}={\bar{\eta }}_{1}+\beta x=\left({\bar{\eta }}_{1},\beta \right)f(x)=(f(x{)}^{T}⊗I_{k_{1}})\mathrm{vec}\left({\bar{\eta }}_{1},\beta \right),$$

where $f(x{)}^{T}=(1,x^{T})\in R^{1\times (p+1)}$. Then, in matrix form,

(A18) $$\eta_{x}=\left(\begin{array}{c} {\bar{\eta }}_{1}+\beta x\\ {\bar{\eta }}_{2} \end{array}\right)=\left(\begin{array}{cc} f(x{)}^{T}⊗I_{k_{1}} & 0\\ 0 & I_{k_{2}} \end{array}\right)\left(\begin{array}{c} \mathrm{vec}\left({\bar{\eta }}_{1},\beta \right)\\ {\bar{\eta }}_{2} \end{array}\right)=F(x)\xi ,$$

where

$$F(x)=\left(\begin{array}{cc} f(x{)}^{T}⊗I_{k_{1}} & 0\\ 0 & I_{k_{2}} \end{array}\right)\in R^{k\times (k_{1}(p+1)+k_{2})},$$

and $\xi =({\bar{\eta }}_{1}^{T},{\mathrm{vec}}^{T}(\beta ),{\bar{\eta }}_{2}^{T}{)}^{T}$ is the vector of parameters of model (13). Note that F(x)ξ∈H for any value of *ξ* with *H* defined in Equation  (10). This notation allows to simplify the expression for the log-likelihood function in Equation (15), and replace it with

(A19) $$m_{\xi }(z)=T(y{)}^{T}F(x)\xi -\psi (F(x)\xi ).$$

The regularity conditions required to derive consistency and asymptotic distribution of the MLE are: For any *ξ* and any $\bar{\eta }$ and for any compact $K\subset \Xi =R^{k_{1}}\times R^{k_{1}p}\times H_{2}$.

(A20) $$\begin{array}{rl} & \mathrm{pr}\left(F(X)\xi =\bar{\eta }\right)<1. \end{array}$$

(A21) $$\begin{array}{rl} & E\left[∥T(Y{)}^{T}F(X)∥\right]<\infty ,\;E\left[\underset{\xi \in K}{\sup}|\psi (F(X)\xi )|\right]<\infty , \end{array}$$

(A22) $$\begin{array}{rl} & E\left[∥T(Y{)}^{T}F(X){∥}^{2}\right]<\infty ,\;E\left[\underset{\xi \in K}{\sup}∥\nabla \psi (F(X)\xi )F(X){∥}^{2}\right]<\infty , \end{array}$$

(A23) $$\begin{array}{rl} & E\left[\underset{\xi \in K}{\sup}∥F(X{)}^{T}\nabla^{2}\psi (F(X)\xi )F(X)∥\right]<\infty . \end{array}$$

To prove the existence, uniqueness and consistency of the MLE under the present model, ${\hat{\xi }}_{n}$, we next show that the assumptions stated in Proposition A.1 are satisfied. The strict convexity of *ψ* implies that, for each fixed *z*, $m_{\xi }(z)$ is a strictly concave function in $\xi \in \Xi =R^{k_{1}+pk_{1}}\times H_{2}$. The concavity of $m_{\xi }(z)$ is preserved under expectation, thus $M(\xi )=Pm_{\xi }$ is concave. The identifiability condition satisfied by the exponential family in Section 3.1 allows applying Lemma 5.35 of van der Vaart [14, p.62] to conclude that

(A24) $$E\left[T(Y{)}^{T}F(Y)\xi -\psi (F(X)\xi )|X\right]\leq E\left[T(Y{)}^{T}F(X)\xi_{0}-\psi (F(X)\xi_{0})|X\right].$$

Taking expectation with respect to *X*, we conclude that $Pm_{\xi }\leq Pm_{\xi_{0}}$ for any *ξ*. Moreover, if $Pm_{\xi_{1}}=Pm_{\xi_{0}}$, $\mathrm{pr}(F(X)\xi_{1}=F(X)\xi_{0})=1$, which contradicts the hypothesis (A20). Finally, the integrability of Equation (A1) follows from Equation  (A21). The conditions A.2–A.4 required by van der Vaart's Theorem 5.23 to derive the asymptotic distribution of M-estimators are easily verifiable under the integrability assumptions stated in Equations (A22) and (A23). The second derivative matrix of $Pm_{\xi }$ at $\xi_{0}$ is

(A25) $$V_{\xi_{0}}=-E\left[F(X{)}^{T}\nabla^{2}\psi (F(X)\xi_{0})F(X)\right].$$

Finally, observe that

$$\begin{array}{rl} P\left[{\dot{m}}_{\xi_{0}}{\dot{m}}_{\xi_{0}}^{T}\right] & =E\left[F(X{)}^{T}\left\{T(Y)-\nabla^{T}\psi (F(X)\xi_{0})\right\}\left\{T(Y{)}^{T}-\nabla \psi (F(X)\xi_{0})\right\}F(X)\right]\\ & =E\left\{F(X{)}^{T}\nabla^{2}\psi (F(X)\xi_{0})F(X)\right\} \end{array}$$

to deduce that, according to the general formula for the asymptotic variance of an M-estimator in (A15), the asymptotic variance of $\sqrt{n}({\hat{\xi }}_{n}-\xi_{0})$ is given by (16).

Lemmas A.5–A.10 and Corollary A.6 are required to prove Proposition 3.2.

Lemma A.5 Assume that $\beta_{01}\in R_{\mathrm{first},d}^{k_{1}\times r}$ and can be written as in Equation (21). Let $(S_{0},T_{0})\in R_{d}^{k_{1}\times d}\times R_{d}^{d\times r}$ with $S_{0}T_{0}=\beta_{01}$. Then, there exists $U\in R_{d}^{d\times d}$ so that $S_{0}=S_{0}(U)$ and $T_{0}=T_{0}(U),$ with

(A26) $$S_{0}(U):=\left(\begin{array}{c} U\\ A_{0}U \end{array}\right)\;\text{and}\;T_{0}(U):=U^{-1}B_{0}.$$

Proof. Let

$$S_{0}=\left(\begin{array}{c} S_{01}\\ S_{02} \end{array}\right),$$

with $S_{01}\in R^{d\times d}$ and $S_{02}\in R^{(k_{1}-d)\times d}$. The matrix $S_{01}T_{0}$ is comprised of the first *d* rows of $\beta_{01}$ which are linearly independent. Then, $d=\mathrm{rank}(S_{01}T_{0})\leq \mathrm{rank}(S_{01})\leq d$, hence $S_{01}$ is invertible. Take $U=S_{01}$. From the expression (21) for $\beta_{01}$, we have

$$\begin{array}{rl} S_{01}T_{0} & =B_{0}\\ S_{02}T_{0} & =A_{0}B_{0}. \end{array}$$

Thus, $T_{0}=U^{-1}B_{0}$, and since $T_{0}T_{0}^{T}\in R^{d\times d}$ and rank *d*,

$$S_{02}=A_{0}B_{0}T_{0}^{T}(T_{0}T_{0}^{T}{)}^{-1}=A_{0}B_{0}B_{0}^{T}U^{-1}(U^{-1}B_{0}B_{0}^{T}U^{-1}{)}^{-1}=A_{0}U.$$

Corollary A.6 Let $\beta_{01}\in R_{\mathrm{first},d}^{k_{1}\times r}$ and can be written as in Equation (21). For *U* in $R_{d}^{d\times d}$ and $S_{0}(U),T_{0}(U)$ as defined in Equation (A26), the pre-image of $\xi_{0}$ through *g*, $g^{-1}(\xi_{0}),$ satisfies

(A27) $$g^{-1}(\xi_{0})≅\left\{\left({\bar{\eta }}_{01},S_{0}(U),T_{0}(U),\beta_{02},{\bar{\eta }}_{02}\right):U\in R_{d}^{d\times d}\right\}.$$

Lemma A.7 $R_{d}^{d\times m}$ with d≤m is an open set in $R^{d\times m}$.

Proof. We will show that the complement of $R_{d}^{d\times m}$ is closed. Consider $(T_{n}{)}_{n\geq 1}\in R^{d\times m}$, each $T_{n}$ of rank strictly less than *d*, and assume that $T_{n}$ converges to $T\in R^{d\times m}$. Note that, $|T_{n}T_{n}^{T}|=0$ for all n≥1, so that $\left|TT^{T}\right|$ is also equal to zero. Hence, rank(T)<d.

Lemma A.8 Θ and S are open sets.

Proof. Up to a homeomorphism, Θ and S are equivalent to

$$R^{k_{1}}\times R_{d}^{k_{1}\times d}\times R_{d}^{d\times r}\times R^{k_{1}\times (p-r)}\times H_{2}\;\text{and}\;R^{k_{1}}\times R^{(k_{1}-d)\times d}\times R_{d}^{d\times r}\times R^{k_{1}\times (p-r)}\times H_{2},$$

respectively, which are products of open sets by Lemma A.7.

Lemma A.9 h:S→M is one to one bicontinuous.

Proof. It suffices to note that $h_{1}:R^{(k_{1}-d)\times d}\times R_{d}^{d\times r}\mapsto R_{\mathrm{first},d}^{k_{1}\times r}$ with

$$(A,B)\to \left(\begin{array}{c} B\\ AB \end{array}\right)$$

satisfies the required properties for *h*. Let $h_{1}^{-1}:R_{\mathrm{first},d}^{k_{1}\times r}\mapsto R^{(k_{1}-d)\times d}\times R_{d}^{d\times r}$ with

(A28) $$\left(\begin{array}{c} B\\ C \end{array}\right)\to (CB^{T}{\left(BB^{T}\right)}^{-1},B).$$

Then, $h_{1}^{-1}(h_{1}(A,B))=(A,B)$ and

$$h_{1}\left(h_{1}^{-1}\left(\begin{array}{c} B\\ AB \end{array}\right)\right)=\left(\begin{array}{c} B\\ AB \end{array}\right),$$

and, therefore, $h_{1}^{-1}$ is the inverse of $h_{1}$. Thus, $h_{1}$ is one to one and bicontinuous.

Lemma A.10 M is open in $\Xi^{\mathrm{res}}$

Proof. It suffices to show $R_{\mathrm{first},d}^{k_{1}\times r}$ is an open set in $R_{d}^{k_{1}\times r}$, or equivalently, that the complement of $R_{\mathrm{first},d}^{k_{1}\times r}$ is a closed set in $R_{d}^{k_{1}\times r}$. Let $\{T_{n}\}\subset R_{d}^{k_{1}\times r}$ be a sequence such that the first *d* rows are not linearly independent and $T_{n}$ converges to $T\in R_{d}^{k_{1}\times r}$. Then, if we write

$$T_{n}=\left(\begin{array}{c} T_{n_{1}}\\ T_{n_{2}} \end{array}\right),$$

with $T_{n_{1}}\in R^{d\times r}$ and $T_{n_{2}}\in R^{(k_{1}-d)\times r}$, we obtain that $|T_{n_{1}}T_{n_{1}}^{T}|=0$ for all *n*. Then $|T_{1}T_{1}^{T}|=0$, where $T_{1}$ comprises of the first *d* rows of *T*, and therefore the first *d* rows of *T* are not linearly independent.

Proof of Proposition 3.2. Proof of Proposition 3.2 We verify that Conditions 2.2, 2.5 and 2.6 are satisfied. Condition 2.2: By Lemma A.8, the set Θ, defined in Equation (20), is open and $\xi_{0}$ belongs to $g(\Theta )=\Xi^{\mathrm{res}}$. Condition 2.5: Since the first *d* rows of $\beta_{01}$ are linearly independent, $\xi_{0}\in M$. Then, applying Lemma A.10 obtains that M is an open set in $\Xi^{\mathrm{res}}$. Consider (S,h) as in Equation (23). By Lemma A.8, S is an open set, and *h* is one-to-one and bicontinuous by Lemma A.9. Furthermore, if

$$s_{0}=({\bar{\eta }}_{01},A_{0},B_{0},\beta_{02},{\bar{\eta }}_{02})\in S,$$

where $A_{0}$ y $B_{0}$ are given in Equation (21), then $h(s_{0})=({\bar{\eta }}_{01},\;\beta_{01},\beta_{02},\;{\bar{\eta }}_{02})=\xi_{0}$. The function *h* is twice continuously differentiable and its Jacobian is full rank. In fact, the latter is

(A29) $$\nabla h({\bar{\eta }}_{1},A,B,\beta_{2},{\bar{\eta }}_{2})=\left(\begin{array}{ccccc} I_{k_{1}} & 0 & 0 & 0 & 0\\ 0 & B^{T}⊗\left(\begin{array}{c} 0\\ I_{k_{1}-d} \end{array}\right) & I_{r}⊗\left(\begin{array}{c} I_{d}\\ A \end{array}\right) & 0 & 0\\ 0 & 0 & 0 & I_{k_{1}(p-r)} & 0\\ 0 & 0 & 0 & 0 & I_{k_{2}} \end{array}\right)$$

of order $\left(k_{1}p+k)\times (d(k_{1}+r-d)+k_{1}(p-r)+k\right)$ with full column rank $d(k_{1}+r-d)+k_{1}(p-r)+k$ (see [21]; also it is a direct implication of Theorem 5 in [22]). Condition 2.6: Consider $\theta_{0}\in g^{-1}(\xi_{0})$, associated with *U*, as shown in Lemma A.5. Since

$$\nabla g({\bar{\eta }}_{1},S,T,\beta_{2},{\bar{\eta }}_{2})=\left(\begin{array}{ccccc} I_{k_{1}} & 0 & 0 & 0 & 0\\ 0 & T^{T}⊗I_{k_{1}} & I_{r}⊗S & 0 & 0\\ 0 & 0 & 0 & I_{k_{1}(p-r)} & 0\\ 0 & 0 & 0 & 0 & I_{k_{2}} \end{array}\right)$$

then,

$$\begin{array}{rl} & \nabla g\left({\bar{\eta }}_{10},\left(\begin{array}{c} U\\ A_{0}U \end{array}\right),U^{-1}B_{0},\beta_{20},{\bar{\eta }}_{20}\right)\\ & \;=\left(\begin{array}{ccccc} I_{k_{1}} & 0 & 0 & 0 & 0\\ 0 & B_{0}^{T}⊗I_{k_{1}} & I_{r}⊗\left(\begin{array}{c} I_{d}\\ A_{0} \end{array}\right) & 0 & 0\\ 0 & 0 & 0 & I_{k_{1}(p-r)} & 0\\ 0 & 0 & 0 & 0 & I_{k_{2}} \end{array}\right)G, \end{array}$$

where

(A30) $$G=\left(\begin{array}{ccccc} I_{k_{1}} & 0 & 0 & 0 & 0\\ 0 & U^{-T}⊗I_{k_{1}} & 0 & 0 & 0\\ 0 & 0 & \left(I_{r}⊗U\right) & 0 & 0\\ 0 & 0 & 0 & I_{k_{1}(p-r)} & 0\\ 0 & 0 & 0 & 0 & I_{k_{2}} \end{array}\right).$$

Since *G* is invertible of order $\left(d(k_{1}+r)+k_{1}(p-r)+k)\times (d(k_{1}+r)+k_{1}(p-r)+k\right)$,

(A31) $$\mathrm{span}\nabla g(\theta_{0})=\mathrm{span}\left(\begin{array}{ccccc} I_{k_{1}} & 0 & 0 & 0 & 0\\ 0 & B_{0}^{T}⊗I_{k_{1}} & I_{r}⊗\left(\begin{array}{c} I_{d}\\ A_{0} \end{array}\right) & 0 & 0\\ 0 & 0 & 0 & I_{k_{1}(p-r)} & 0\\ 0 & 0 & 0 & 0 & I_{k_{2}} \end{array}\right).$$

Next, since $\nabla h(s_{0})=\nabla g(\theta_{0})G^{-1}\tilde{I}$, with

$$\tilde{I}=\mathrm{diag}\left(\begin{array}{ccccc} I_{k_{1}} & I_{d}⊗\left(\begin{array}{c} 0\\ I_{k_{1}-d} \end{array}\right) & I_{rd} & I_{k_{1}(p-r)} & I_{k_{2}} \end{array}\right),$$

we have $\mathrm{span}\nabla h(s_{0})\subset \mathrm{span}\nabla g(\theta_{0})$. Applying again Theorem 5 in [22] obtains $\mathrm{rank}(\nabla g(\theta_{0}))=d(k_{1}+r-d)+k_{1}(p-r)+k=\mathrm{rank}(\nabla h(s_{0}))$. Therefore, $\mathrm{span}\nabla h(s_{0})=\mathrm{span}\nabla g(\theta_{0})$.

Proof of Theorem 3.3. Proof of Theorem 3.3 In Theorem 3.1 we verified that the conditions of Theorem 5.23 in [14] are satisfied for the maximum likelihood estimator under model (12) satisfying (13). The asymptotic variance of the MLE estimator is given in Equation (16). We also showed Conditions 2.2–2.6 are satisfied in the proof of Proposition 3.2. The result follows from Proposition 2.7 since $-V_{\xi_{0}}=W_{\xi_{0}}^{-1}$ and

$$\begin{array}{rl} \text{avar}\{\sqrt{n}({\hat{\xi }}_{n}^{\mathrm{res}}-\xi_{0})\} & =\Pi_{\xi_{0}(W_{\xi_{0}}^{-1})}W_{\xi_{0}}\Pi_{\xi_{0}(W_{\xi_{0}}^{-1})}^{T}\\ & =\nabla g(\theta_{0})(\nabla g(\theta_{0}{)}^{T}W_{\xi_{0}}^{-1}\nabla g(\theta_{0}){)}^{†}\nabla g(\theta_{0}{)}^{T}\\ & =\Pi_{\xi_{0}(W_{\xi_{0}}^{-1})}W_{\xi_{0}}. \end{array}$$

Now, by Equation (21),

$$C_{0}=\left(\begin{array}{c} I_{d}\\ A_{0} \end{array}\right),$$

$\mathrm{span}\nabla g(\theta_{0})$ in Equation (A31) is equal to span(G), with *G* defined in Equation (26). The result follows if we prove that span(G) does not depend on the decomposition of $\beta_{01}$. Suppose that $\beta_{01}=C_{1}B_{1}$. Then $C_{1}=C_{0}M$ and $B_{1}=M^{-1}B_{0}$, for an invertible $M\in R^{d\times d}$. Let $G_{1}$ be the matrix corresponding to *G* using the new decomposition of $\beta_{01}$. Then, $G_{1}=GH$, with

$$H=\left(\begin{array}{ccccc} I_{k_{1}} & 0 & 0 & 0 & 0\\ 0 & M^{-T}⊗I_{k_{1}} & I_{r}⊗M & 0 & 0\\ 0 & 0 & 0 & I_{k_{1}(p-r)} & 0\\ 0 & 0 & 0 & 0 & I_{k_{2}} \end{array}\right),$$

where *H* is invertible. Therefore, $\mathrm{span}(G_{1})=\mathrm{span}(G)$ and the result follows.
